# Time Trends and Inequalities of Under-Five Mortality in Nepal: A Secondary Data Analysis of Four Demographic and Health Surveys between 1996 and 2011

**DOI:** 10.1371/journal.pone.0079818

**Published:** 2013-11-04

**Authors:** Chandrashekhar T. Sreeramareddy, H. N. Harsha Kumar, Brijesh Sathian

**Affiliations:** 1 Department of Population Medicine, Faculty of Medicine and Health Sciences, University Tunku Abdul Rahman, Bandar Sungai Long, Selangor, Malaysia; 2 Department of Community Medicine, Kasturba Medical College, Mangalore, Karnataka, India; 3 Department of Community Medicine, Manipal College of Medical Sciences, Pokhara, Western Development Region, Nepal; Indiana University, United States of America

## Abstract

**Background:**

Inequalities in progress towards achievement of Millennium Development Goal four (MDG-4) reflect unequal access to child health services.

**Objective:**

To examine the time trends, socio-economic and regional inequalities of under-five mortality rate (U5MR) in Nepal.

**Methods:**

We analyzed the data from complete birth histories of four Nepal Demographic and Health Surveys (NDHS) done in the years 1996, 2001, 2006 and 2011. For each livebirth, we computed survival period from birth until either fifth birthday or the survey date. Using direct methods i.e. by constructing life tables, we calculated yearly U5MRs from 1991 to 2010. Projections were made for the years 2011 to 2015. For each NDHS, U5MRs were calculated according to child's sex, mother’s education, household wealth index, rural/urban residence, development regions and ecological zones. Inequalities were calculated as rate difference, rate ratio, population attributable risk and hazard ratio.

**Results:**

Yearly U5MR (per 1000 live births) had decreased from 157.3 (95% CIs 178.0-138.9) in 1991 to 43.2 (95% CIs 59.1-31.5) in 2010 i.e. 114.1 reduction in absolute risk. Projected U5MR for the year 2015 was 54.33. U5MRs had decreased in absolute terms in all sub groups but relative inequalities had reduced for gender and rural/urban residence only. Wide inequalities existed by wealth and education and increased between 1996 and 2011. For lowest wealth quintile (as compared to highest quintile) hazard ratio (HR) increased from 1.37 (95% CIs 1.27, 1.49) to 2.54 ( 95% CIs 2.25, 2.86) and for mothers having no education (as compared to higher education) HR increased from 2.55 (95% CIs 1.95, 3.33) to 3.75 (95% CIs 3.17, 4.44). Changes in regional inequities were marginal and irregular.

**Conclusions:**

Nepal is most likely to achieve MDG-4 but eductional and wealth inequalities may widen further. National health policies should address to reduce inequalities in U5MR through ‘inclusive policies'.

## Introduction

Globally, 9-10 million children die each year before reaching their fifth birthday[1]. Under-five mortality rate (U5MR), a very sensitive indicator of population health has been adopted as one of the eight Millenium Development Goals (MDGs) by members of United Nations in 2000[2]. A reduction of U5MR will reflect the impact of child survival interventions and global health policies aimed at international development[3].The fourth MDG (MDG-4) targets a two-thirds reduction in the 1990 U5MR by 2015[2]. Despite a steady progress made towards achieving MDG-4 in many countries[4,5], decline of U5MR in sub-Saharan Africa and some Asian countries has been slow[6] and socio-economic inequalities exist in terms mortality as well as coverage indicators of child survival interventions[4,7–9]. The countdown to 2015 was conceived in 2003 for tracking global and country-level progress towards achievement of MDGs four and five aims to monitor coverage of effective interventions in countries with high child mortality rates[10]and emphasises on the need to address the inequalities in service coverage indicators and child mortality rates[9–11].

Nepal, a small landlocked country located on the lap of Himalayan mountain range has a diverse topography and a multi-ethnic population. Despite a decade long armed conflict and ensuing political instability, Nepal has achieved a remarkable progress in public health and has reduced U5MR from 158 (per 1000 live births) in 1990 to 54.4 in 2011[12–14]. A prediction by exponential-decline regression curves using Nepal Demographic Health Survey (NDHS) data, has shown that Nepal may achieve U5MR of 38 during the period 2011–2015[15]. Analyses of three NDHS and two Nepal Living Standards Survey(NLSS) (both data up to 2006) have projected that U5MR would drop to 31 by 2015[16]. Nepal's success towards likely achievement of MDG-4 can be attributed to several child survival interventions implemented by the Government of Nepal(GoN). These are expanded program on immunization, vertical disease control programs for childhood diarrhea and pneumonia, integrated management of childhood illness(IMCI), safe motherhood program, traditional birth attendants(TBAs), female community health volunteers(FCHVs)[17] in addition to more recently implemented community-based IMCI, 'Aama Surckchhya Karayakram' (to improve facility-based births) and Community-based Newborn Care Package (CB-NCP)[13,18].

Though MDG-4 target is likely to be achieved in Nepal by 2015[15,18] a national level indicator (U5MR) may have masked inequalities existing among population sub groups and at sub national regions. Apart from the cross-national reports[7,8,19,20], studies from South Africa, Mozambique, Uganda, Zimbabwe, Indonesia, Sri Lanka, Papua New Guinea and India have reported that inequalities exist in U5MR and underscored the importance of monitoring inequalities, improving service coverage, and up-scaling child survival interventions[21–28]. Analyses of inequalities in U5MR will identify the population sub groups and sub national regions that are not covered by child survival interventions. Information about inequalities in U5MR will help the policy makers to improve child survival by setting up strategies to reduce inequalities[11].Health disparities in Nepal according to socio-economic and sub national regions are well known[29,30]. In this paper, we are reporting trends of yearly U5MR in Nepal from 1991 to 2010 based on data from four NDHSs and projected U5MR for the year 2015 i.e. achieving MDG-4.We also estimated inequalities in U5MR according to social and economic sub groups and sub national regions.

## Methods

### Ethics Statement

Standard protocols, data collection tools and procedures for NDHSs were reviewed and approved by Independent Review Boards(IRBs) of New ERA(non-governmental organisation) and ORC Macro International. The survey participants were informed that their participation was voluntary and assured about confidentiality of the information they will provide. The participants could abstain from responding to any of the questions. Prior to the interview, details of the survey were explained to each participant and informed consent was obtained. Written consent was not necessary since participants were not subjected to any type of intervention. Interviewer recorded the consent in the questionnaire and signed the consent form. IRBs of both ORC Macro International and New ERA approved this consent procedure.

### Data sources

We used the raw data of complete birth histories collected from nationally representative samples of ever-married women aged 15-49 years during four NDHSs done in the years 1996, 2001, 2006 and 2011. The samples of ever-married women surveyed in these four NDHSs were 8580, 8885, 10973 and 12918 respectively. Response rates for all four surveys were 98% and above. During each survey, ever-married women were interviewed to collect information about complete birth history in the women’s questionnaire. For each live births, following details about birth history were used for our analysis; date of birth, if the child was ‘dead’ or ‘alive’; if ‘dead’, age at death, date of death and date of interview (to compute survival period for each live birth). NDHSs had also collected data about household demographics, ownership of household assets, dwelling characteristics, health and nutritional status of women and children, coverage of healthcare services for maternal and child health, current knowledge and practices related to health and healthcare seeking behavior. 

### Markers of inequality

**Figure 1:**
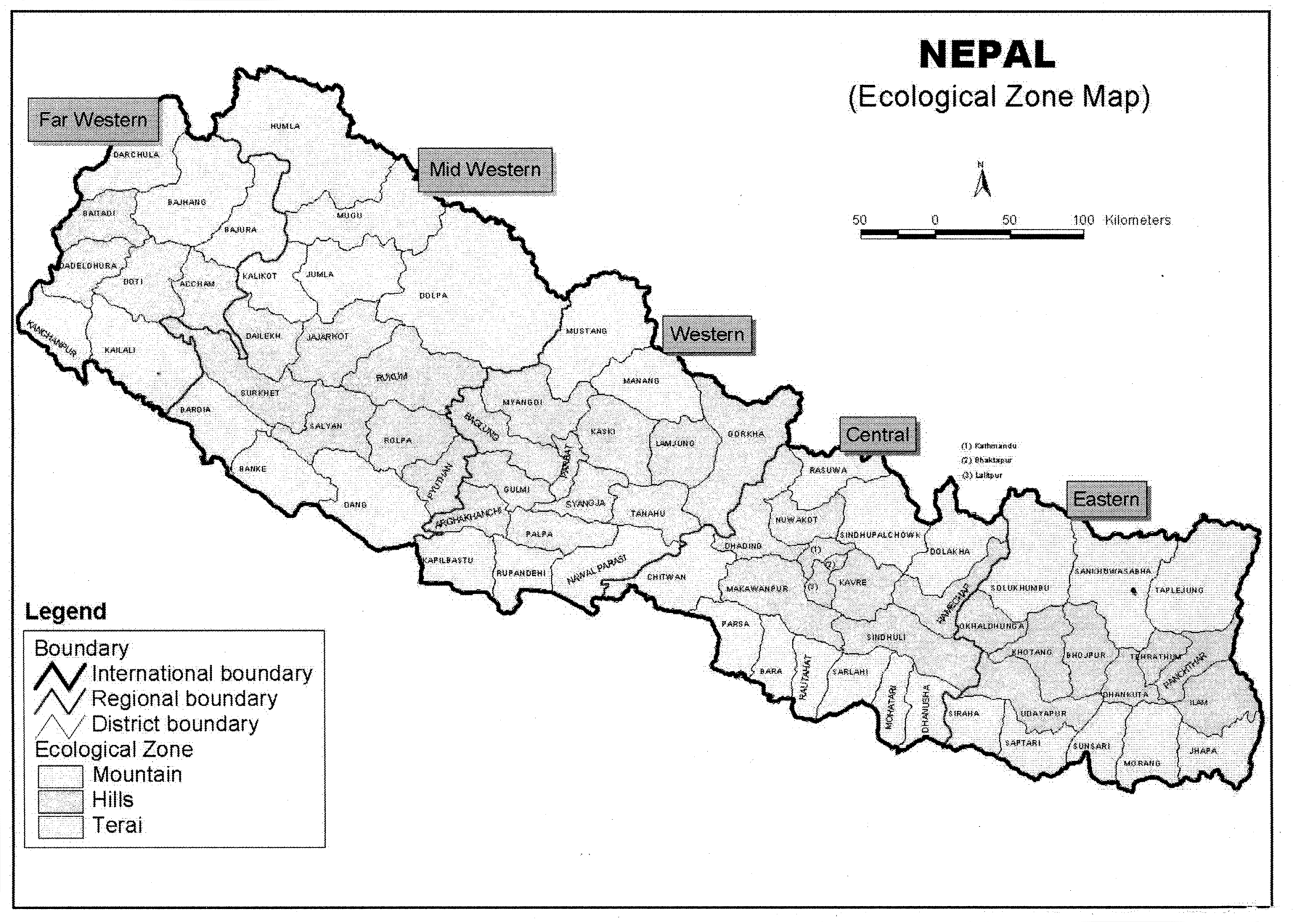
Map of Nepal showing development regions and ecological zones.

Child's sex was included as a marker of inequality since gender inequality in health are well known in Nepal and other Asian countries[31]. Mother’s highest level of education was classified into four groups:1) ‘no education’, 2)‘up to primary’, 3)‘up to secondary’ and 4) ‘tertiary/higher’. Wealth index which is a relative index of household wealth was calculated based on a standard set of household assets, dwelling characteristics and ownership of consumer items as observed by the interviewers. Each woman was ranked based on a household asset score and was assigned to wealth quintiles. Accordingly, first quintile was poorest 20% of the households and fifth quintile was wealthiest 20% of the households[32]. Despite its limitations, wealth index has been accepted as a fairly good measure of economic status and used a proxy indicator for income[33]. Administratively, Nepal is divided into five developmental regions i.e. Eastern, Central, Western, Mid-western and Far-western. Rural/urban residence was also included as marker of inequality. Topographically, Nepal is divided into three ecological zones i.e. mountain, hill and plains/terai (Figure 1). Developmental regions and ecological zones were used to examine differences, if any in U5MR, since the degree of development in Nepal varies according to these two types of sub national regions and rural/urban residence. 

### Statistical analysis

Using complete birth history of each live birth, we calculated survival period from birth until fifth birthday or the date of interview or until death, if the baby was dead. For calculating U5MR, we used direct methods recommended by the World Bank. This method adopts survival/life table analysis to calculate mortality rates according to different periods of risk. We merged the data on complete birth histories of four surveys and regrouped it by each year from 1991 to 2010. On STATA-10, we calculated yearly U5MR by constructing a separate life table for each year. We constructed a line diagram on Microsoft excel using yearly U5MR estimates and their 95% confidence intervals (95% CIs). We also constructed a scatter plot, and added a trend line. We performed correlation between time (in years) and yearly U5MR to calculate R^2^ value (correlation coefficient) and derived a regression equation from log-linear regression analysis. To make projection towards achieving MDG-4 target, Poisson regression analysis for count data was done to calculate predicted U5MRs for the years 2011 to 2015 according to the methods described by Rosenberg[34] (Table S1). For each NDHS, U5MRs for each of the socio-economic and regional sub groups were calculated using survival/life table analysis as explained above. 

U5MRs for sub groups in each NDHS were used to calculate various measures of inequalities, such as rate difference (RD), rate ratio (RR), population attributable risk (PAR), univariate and multivariate hazard ratio (HR) as recommended by Schneider et al. [35]. According to Schneider, higher value of RR and RD suggest higher inequality. PAR is a measure of potential impact on population health and also known as aetiologic fraction. Hazard ratio (HR), slope index of inequality (SII) and relative index of ineqaulity (RII) are other measures of potential impact on population health[35]. Researchers have emphasised that all measures of inequalities should be reported as interpretations of absolute (RD & RR) and relative measures of inequalities (PAR, HR, RII, SII etc) may be different[36,37].

To calculate RD and RR, we considered the highest and lowest U5MR in each sub groups. For PAR, we used overall U5MR for the given survey and highest U5MR in a sub group. For each survey, measures of inequalities were calculated for following sub groups: child's sex, mother's education, wealth index, rural/urban residence, development regions and ecological zones. Aggregate data of U5MR was used to calculate SII and RII for mother's education and wealth index since they can be calculated for ordinal variables only. SII and RII were caculated by weighted least squares regression after arranging cumulative distribution of live births in a descending order of an ordinal variable[35]. Univariate HRs and 95% CIs were calculated for each sub group in STATA-10 using *svy* command and sample weights were included to account for sampling design of NDHS. To assess the extent to which observed inequalites may have been affected by other socio-economic and regional factors, we performed Cox proportional regression analyses to calculate HR and their 95% CIs. All population sub groups used in analysis of inequalities were included into Cox regression analyses on IBM SPSS version 20. *Complex samples analysis* option was used to account for multistage sampling design adopted in NDHS. For all the statistical analysis, a p-value <0.05 was considered as statistically significant. 

## Results

### Trends and projection of U5MR in Nepal

**Figure 2:**
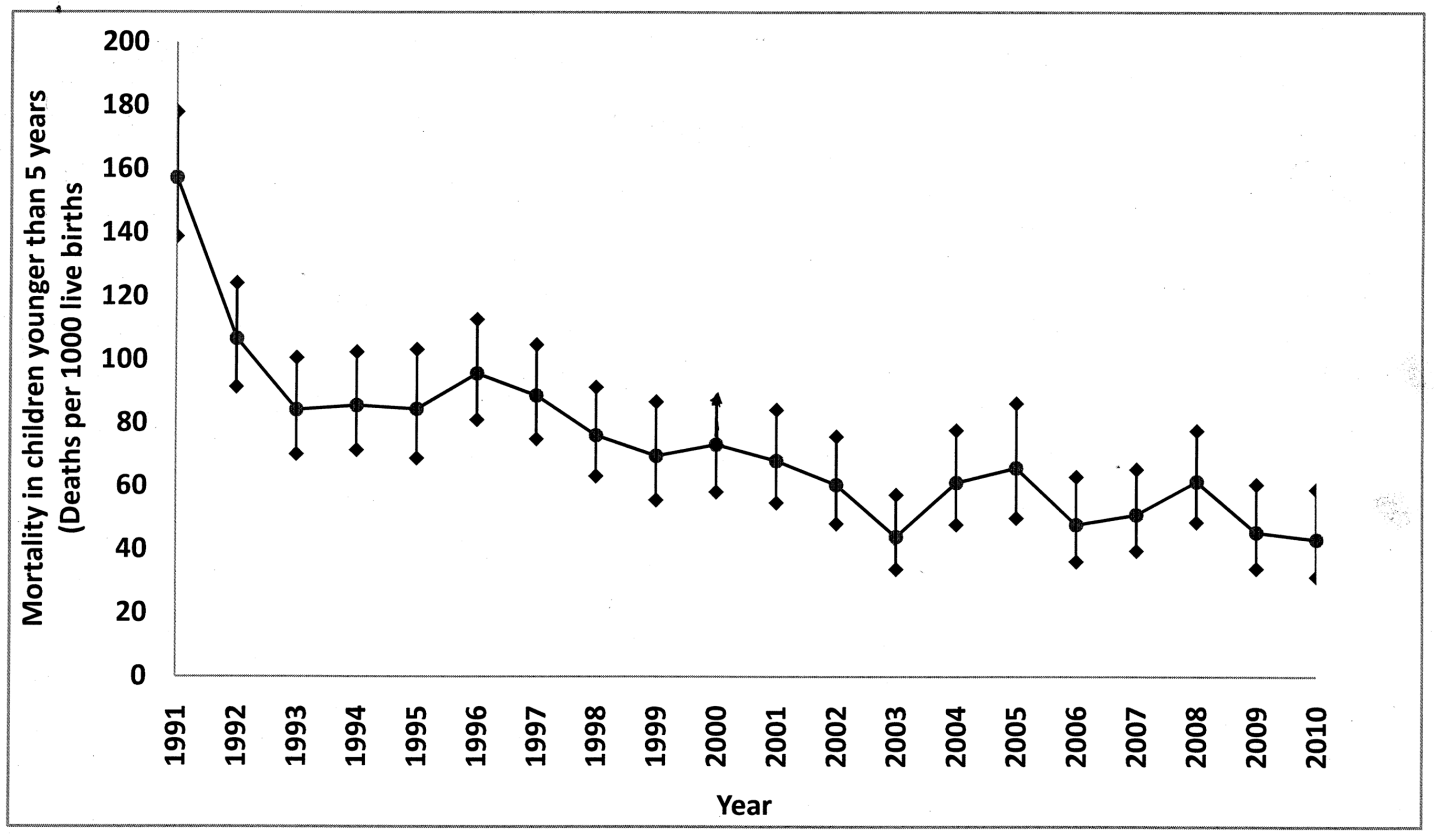
Yearly U5MR in Nepal from 1991 to 2010.

**Figure 3:**
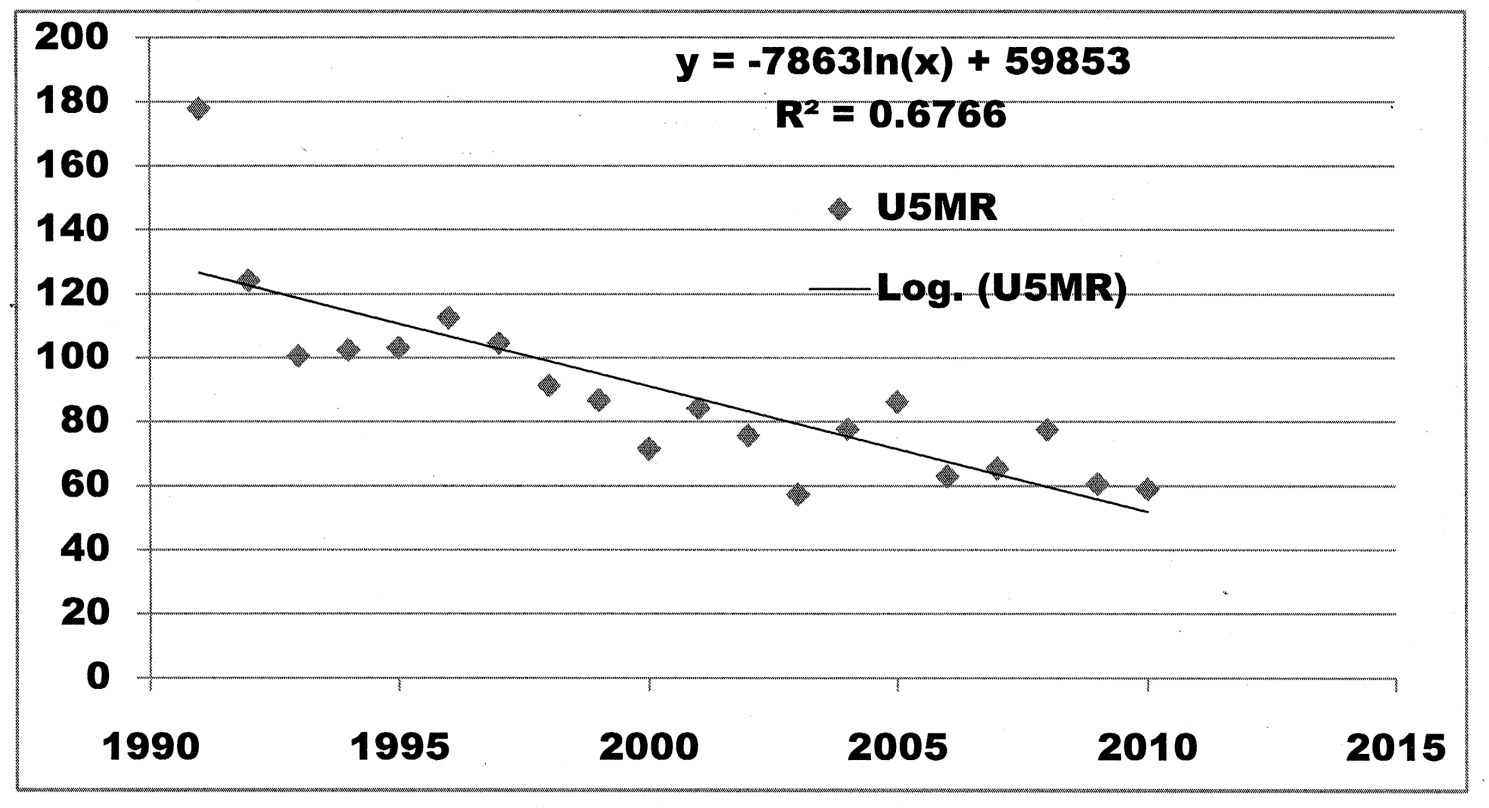
Scatter plot and trend line for loglinear regression analysis for relationship between time periods 1991 to 2010 and yearly U5MR.

Yearly U5MRs showed that U5MR in Nepal had decreased steadily between 1991 and 2010 (Figure 2) from 153.7 in 1991 to 43.2 in 2010 accounting for 72.5% decline in 20 years. Yearly decline in U5MR was 4.21% whereas average decline for each five-year period (the time interval at which NDHS were carried out) was 19.4%. U5MR in 1994 (mid-point of five years prior to NDHS-1996) was 85.6 (95% CIs 102.5-71.5) whereas U5MR in 2008 (mid-point of five years prior to NDHS-2011) was 61.6 (95% CIs 77.7-48.8)corrresponding to a change of 22.6 in absolute risk or 36.7% decline in U5MR between NDHS1996 and NDHS 2011. By loglinear regression analysis for trend, correlation coefficient (R^2^) was 0.6766 (Figure 3) which means that about 68% variation in U5MR is explained by variation in time. Projected U5MRs for the years 2011, 2012, 2013, 2014 and 2015 were 64.5, 61.8, 59.2, 56.7 and 54.3 respectively (Table S1).

### Inequalities in U5MR according to various sub groups

Number of births and under-five child deaths in each survey revealed that inequalities were present according to sub groups. In all four NDHSs, more than 80% of the births and deaths had occured among mothers with no education (Tables 1 and 2) and women from poorer and poorest households accounted for nearly 50% of births and more than 50% of under-five child deaths. Nearly 50% of under-five child deaths were occuring in the mountain region (Table 2). 

**Table 1:** Socio-demographic characteristics of the women in the four NDHS samples from 1996 to 2011.

Distribution of sample	NDHS 1996 N (%)	NDHS 2001 N (%)	NDHS 2006 N (%)	NDHS 2011 N (%)
Total sample size	8429	8726	10793	12674
**Mother's education**				
Higher	112 (1.3)	112 (1.3)	481 (4.5)	1064 (8.4)
Secondary	688 (8.2)	1071 (12.3)	2727 (25.3)	4584 (36.2)
Primary or less	893 (10.6)	1274 (14.6)	1908 (17.7)	2149 (17)
No education	6736 (79.9)	6269 (71.8)	5677 (52.6)	4876 (38.5)
**Wealth quintiles**				
Highest (richest	1711 (20.3)	1876 (21.5)	2340 (21.7)	3080 (24.3)
Fourth (richer)	1623 (19.3)	1708 (19.6)	2267 (21)	2516 (19.9)
Middle	1760 (20.8)	1569 (18.0)	1974 (18.3)	2336 (18.4)
Second (poorer)	1585 (18.8)	1711 (19.6)	2005 (18.6)	2296 (18.1)
Lowest (poorest)	1750 (20.8)	1826 (21.3)	2207 (20.4)	2446 (19.3)
**Development regions**				
Eastern	1683 (20)	2068 (23.7)	2529 (23.4)	3019 (23.8)
Central	2515 (29.8)	2392 (27.4)	2739 (25.4)	3009 (23.7)
Western	1594 (18.9)	1556 (17.8)	2105 (19.5)	2304 (18.2)
Midwestern	1390 (16.5)	1142 (13.1)	1691 (15.7)	2275 (18)
Far western	1247 (14.8)	1568 (18)	1729 (16)	2067 (16.3)
**Urban/rural Residence**				
Urban	954 (11.3)	1154 (13.2)	2949 (27.3)	3701 (29.2)
Rural	7475 (88.7)	7572 (86.8)	7844 (72.7)	8973 (70.8)
**Ecological Zone**				
Terai	3771 (44.7)	4295 (49.2)	5084 (47.1)	5667 (44.7)
Hill	3597 (42.7)	3243 (37.2)	4229 (39.2)	4974 (39.2)
Mountain	1061 (12.6)	1188 (13.6)	1480 (13.7)	2033 (16)

**Table 2:** Total births and deaths among children aged less than five years according sub groups in four NDHSs from 1996 to 2011.

**Sub group**	NDHS1996		NDHS 2001		NDHS 2006		NDHS 2011	
	Deaths (%)	Births (%)	Deaths (%)	Births (%)	Deaths (%)	Births (%)	Deaths (%)	Births (%)
Total events	2489	22786	2190	24878	3386	25475	4089	25064
**Mother’s education**								
Higher	12 (0.5)	336 (1.5)	25 (1.1)	743 (9.9)	5 (0.1)	163 (1.6)	4 (0.1)	161 (0.6)
Secondary	113 (4.5)	2601 (11.4)	214 (9.8)	4499 (18.1)	116 (3.4)	1942 (7.6)	84 (2.1)	1274 (5.1)
Primary	239 (9.6)	3056 (13.4)	307 (14.0)	4211 (16.9)	242 (7.2)	2895 (11.4)	226 (5.5)	2046 (8.2)
No education	2125 (85.4)	16793 (73.6)	1643 (75.1)	15420 (62.0)	3023 (89.3)	20475 (80.4)	3775 (92.3)	21583 (86.1)
**Household wealth index**								
Highest	266 (10.7)	3901 (17.1)	254 (11.6)	4569 (18.4)	503 (14.8)	5073 (19.9)	499 (12.2)	4671 (18.6)
Fourth	407 (16.4)	4446 (19.5)	351 (16.1)	4516 (18.2)	595 (17.6)	4809 (18.9)	711 (17.3)	4752 (19.0)
Middle	523 (21.0)	4323 (19.0)	410 (18.7)	4626 (18.6)	605 (17.9)	4676 (18.4)	931 (22.8)	5081 (20.3)
Second	541 (21.7)	4629 (20.3)	478 (21.8)	4962 (19.9)	735 (21.7)	5014 (19.7)	902 (22.1)	4802 (19.2)
Lowest	752 (30.2)	5487 (241.1)	697 (31.8)	6205 (25.0)	948 (28.0)	5903 (23.2)	1046 (25.6)	5758 (23.0)
**Rural/urban residence**								
Urban	451 (18.1)	5436 (23.9)	444 (20.2)	6136 (24.7)	318 (9.4)	3126 (12.3)	286 (7.0)	2582 (10.3)
Rural	2038 (81.9)	17350 (76.1)	1746 (79.8)	18742(75.3)	3068 (90.6)	22349 (87.7)	3803 (93.0)	22482 (89.7)
**Developmental region**								
Eastern	419 (16.8)	4906 (21.5)	393 (17.9)	5419 (21.8)	679 (20.0)	5993 (23.5)	684 (16.7)	4907 (19.6)
Central	635 (25.5)	5906 (25.9)	481 (22.0)	5856 (23.5)	945 (27.9)	6871 (27.0)	1113 (27.3)	7231 (28.9)
Western	407 (16.4)	4201 (18.4)	293 (13.4)	4255 (17.1)	500 (14.8)	4391 (17.2)	672 (16.4)	4744 (18.9)
Mid-western	547 (22.0)	3877 (17.0)	525 (24.0)	4851 (19.5)	483 (14.3)	3401 (13.4)	847 (20.7)	4275 (17.1)
Far-western	481 (19.3)	3896 (17.1)	498 (22.7)	4497 (18.1)	779 (23.0)	4819 (18.9)	773 (18.9)	3907 (15.6)
**Ecological region**								
Terai (plains)	411 (16.5)	3168 (13.9)	455 (20.8)	4436 (17.8)	639 (18.9)	3609 (14.2)	701 (17.1)	3262 (13.0)
Hill	838 (33.7)	8590 (37.7)	812 (37.1)	9760 (39.2)	1075 (31.7)	9201 (36.1)	1558 (38.1)	10539 (42.0)
Mountain	1240 (49.8)	11028 (48.4)	923 (42.1)	10682(42.9)	1672 (49.4)	12665 (49.7)	1830 (44.8)	11263 (44.9)

Between 1996 and 2011a consistent decline in U5MR was seen in all subgroups (Table 3). A few notable exceptionswere a slight increase in U5MR among children born to mothers educated up to secondary school, from 52.4 in 1996 to 56.9 in 2001, followed by an increase from 32.0 in 2006 to 43.0 in 2011. Other exceptions were among under-five children born in richer (fourth quintile) households i.e. an increase from 40.9 in 2006 to 50.6 in 2011; children born in urban areas (between 2001 and 2006) and children born in western development region (between 2001 and 2006) (Table 3).

**Table 3:** Overall U5MRs and inequalities of U5MR according to socio-economic and regional sub groups in four NDHS from 1996 to 2011.

**Sub group**	NDHS1996	NDHS2001	NDHS2006	NDHS2011	% change
Total	118.2	87.0	62.9	53.4	54.8
**Child's sex**					
Male×	113.3	84.2	60.1	53.1	53.1
Female	122.9	89.9	65.8	53.7	56.3
**Rate difference**	**8.7**	**5.7**	**5.7**	**0.6**	
**Rate ratio**	**1.09**	**1.07**	**1.10**	**1.01**	
**PAR**	**4.2**	**3.2**	**4.5**	**0.6**	
**Univariate HR**	**1.04 (1.01, 1.07**)** p=0.018**	**1.05 (1.02, 1.07**)** p=0.005**	**1.03 (0.97, 1.05**)** p=0.622**	**1.01 (1.0, 1.05**)** p=0.044**	
**Mother’s education**					
Higher×	13.4	000*	28.2	28.5	**112.7**
Secondary	52.4	56.9	32.0	43.0	17.9
Primary	107.5	61.8	57.4	53.9	49.9
No education	126.4	97.0	74.8	62.2	50.7
**Rate difference**	**113**	**39.1**	**46.6**	**33.7**	
**Rate ratio**	**9.42**	**0**	**2.65**	**2.18**	
**PAR**	**88.6%**	1%	**55.2%**	**46.6%**	
**Univariate HR**	**1.16 (1.13, 1.19**)** p<0.001**	**1.16 (1.14,1.19**)** p<0.001**	**1.31 (1.27,1.34**)** p<0.001**	**1.26 (1.23, 1.28**)** p<0.001**	
**Household wealth index**					
Highest×	75.8	58.0	45.1	27.3	63.9
Fourth	109.2	82.9	40.9	50.6	53.7
Middle	139.6	87.3	86.5	52.8	62.1
Second	115.4	90.6	61.8	55.7	51.7
Lowest	135.7	104.6	71.4	65.3	51.9
**Rate difference**	**59.9**	**46.6**	**26.3**	**38.0**	
**Rate ratio**	**1.78**	**1.79**	**1.58**	**2.39**	
**PAR**	**35.9%**	**33.0%**	**35%**	**48.9%**	
**Univariate HR**	**0.92 (0.91, 0.95**)** p<0.001**	**0.93 (0.92, 0.94**)** p<0.001**	**0.92 (0.91, 0.93**)** p<0.001**	**0.87 (0.87, 0.89**)** p<0.001**	
**Rural/urban residence**					
Urban×	72.8	49.8	53.7	43.8	39.8
Rural	122.5	90.9	65.7	55.8	54.5
**Rate difference**	**49.7**	**41.1**	**12**	**12**	
**Rate ratio**	**1.69**	**1.83**	**1.22**	**1.27**	
**PAR**	**38.4%**	**42.8%**	**14.6%**	**17.9%**	
**Univariate HR**	**1.20 (1.15, 1.25**)** p<0.001**	**1.27 (1.23, 1.32**)** p<0.001**	**1.09 (1.05, 1.14**)** p=0.007**	**1.19 (1.15, 1.23**)** p<0.001**	
**Developmental region**					
Eastern×	103.1	82.8	51.2	49.4	52.1
Central	99.2	88.9	51.0	50.2	49.4
Western	113.9	58.1	63.3	44.6	60.8
Mid-western	143.1	84.1	79.7	48.9	65.8
Far-western	145.7	114.7	77.1	73.7	49.4
**Rate difference**	**42.6**	**31.9**	**25.9**	**24.3**	
**Rate ratio**	**1.41**	**1.39**	**1.51**	**1.65**	
**PAR**	**16.1%**	**33.2%**	**18.9%**	**16.5%**	
**Univariate HR**	**1.03 (1.02, 1.04**)** p=0.001**	**1.04 (1.03, 1.05**)** p<0.001**	**1.03 (1.01, 1.04**)** p=0.015**	**0.94 (0.92, 0.96**)** p=0.016**	
**Ecological region**					
Terai (Plains)	106.9	85.5	69.4	48.5	54.6
Hill	110.6	77.2	52.3	50.4	54.4
Mountain×	173.4	114.7	70.5	70.0	59.6
**Rate difference**	**66.5**	**29.2**	**1.1**	**21.5**	
**Rate ratio**	**1.62**	**1.33**	**1.02**	**1.45**	
**PAR**	**9.6%**	**11.3%**	**16.9%**	**9.2%**	
**Univariate HR**	**0.93 (0.91, 0.95**)** p<0.001**	**0.99 (0.98, 0.99**)** p<0.001**	**0.99 (0.98, 0.99**)** p=0.01**	**0.94 (0.92, 0.96**)** p<0.001**	

* No deaths were reported in this group in 2001 NDHS

× Indicates reference category for univariate HR

### Child's sex

In 1996, U5MR was higher among girls than the boys (122.9 versus 113.3) and the RD was 8.7 but in 2011 U5MR was slightly higher among girls than the boys (53.7 versus 53.1 ) and the RD was only 0.6. HR for girls had decreased from 1.04 in 1996 to 1.01 in 2011 (Table 3).

### Mother's education

In all NDHSs, U5MR was highest in 'no education' group and lowest in 'higher education' group and there was a clear gradient across the education sub groups. In 1996, U5MR in 'no education' group was 126.4 but it decreased to 62.2 in 2011. Similarly, U5MR had decreased from 107.5 to 53.9 in 'primary education' group. RD in U5MRs between education sub groups was high during all four survey periods though this gap narrowed from 113 in 1996 to 33.7 in 2011. RR and PAR had decreased over this period but was still high in 2011 (RR 2.18 and PAR 46.6%) (Table 3). Though univariate HR increased marginally from 1.16 in 1996 to 1.26 in 2011, HRs by Cox regression increased between 1996 and 2011 in all educational sub groups (Table 4). For example, in 1996 children born to mothers with 'no education' had 2.55 times higher risk of death before five years as compared to children born to mothers with 'higher education', but in 2011 HR increased to 3.75. Between 1996 and 2011, SII had increased whereas RII had decreased for eductaional sub groups (Table S2).

**Table 4:** Cox proportional hazards regression analyses for inequality for each of the four NDHSs from 1996 & 2011*.

**Sub groups**	NDHS 1996		NDHS 2001		NDHS 2006		NDHS 2011	
	HR (95% CIs)	p-value	HR (95% CIs)	p-value	HR (95% CIs)	p-value	HR (95% CIs)	p-value
**Child's sex**								
Male	1		1		1		1	
Female	1.03 (1.00,1.07)	0.013	1.05 (1.02,1.08)	<0.001	1.00 (1.03, 1.05)	0.048	1.01 (1.02, 1.04)	0.041
**Mother’s education**								
Higher	1		1		1		1	
Secondary	1.44 (1.10, 1.89)	<0.001	1.18 (1.08, 1.29)	<0.001	1.26 (1.12, 1.78)	<0.001	1.48 (1.29, 1.69)	<0.001
Primary	1.81 (1.37, 2.39)	<0.001	1.70 (1.22, 2.38)	<0.001	1.74 (1.27, 2.39)	<0.001	2.26 (1.92, 2.66)	<0.001
No education	2.55 (1.95, 3.33)	<0.001	2.35 (1.67, 3.32)	<0.001	2.99(2.12, 4.21)	<0.001	3.75 (3.17, 4.44)	<0.001
**Wealth index**								
Highest	1		1		1		1	
Fourth	1.14 (1.06, 1.27)	<0.001	1.33 (1.22, 1.44)	<0.001	1.23 (1.12, 1.36)	<0.001	1.27 (1.14, 1.41)	<0.001
Middle	1.10 (1.01, 1.20)	<0.001	1.41 (1.29, 1.54)	<0.001	1.37 (1.21, 1.55)	<0.001	1.60 (1.43, 1.78)	<0.001
Second	1.18 (1.09, 1.28)	<0.001	1.39 (1.27, 1.51)	<0.001	1.49 (1.32, 1.69)	<0.001	2.05 (1.84, 2.29)	<0.001
Lowest	1.37 (1.27, 1.49)	<0.001	1.42 (1.30, 1.56)	<0.001	1.75 (1.54, 1.98)	<0.001	2.54 (2.25, 2.86)	<0.001
**Urban/rural residence**								
Urban	1		1		1		1	
Rural	1.17 (1.05, 1.23)	<0.001	1.18 (1.08, 1.29)	<0.001	1.06 (1.05, 1.17)	0.034	1.05 (1.04. 1.15)	<0.001
**Development region**								
Eastern	1		1		1		1	
Central	1.04 (0.95, 1.13)	0.333	0.96 (0.87, 1.05)	0.052	0.97 (0.90, 1.05)	0.052	1.03 (0.92, 1.15)	0.709
Western	0.98 (0.91, 1.08)	0.393	0.97 (0.89, 1.06)	0.080	0.93 (0.86, 1.01)	0.072	1.00 (0.91, 1.11)	0.932
Mid-western	1.02 (0.94, 1.13)	0.469	0.92 (0.83, 1.02	0.074	0.98 (0.89, 1.07)	0.441	0.91 (0.82, 1.01	0.632
Far-western	1.05 (0.97, 1.15)	0.166	1.04 (0.93, 1.17)	0.632	0.96 (0.87, 1.07)	0.134	1.09 (1.00, 1.20)	0.003
**Ecological region**								
Terai (plains)	1		1		1		1	
Hill	1.06 (1.00, 1.13)	0.002	1.12 (1.05, 1.20)	<0.001	1.01 (0.94, 1.09)	0.829	0.92 (0.85, 0.99)	0.001
Mountain	1.13 (1.06, 1.22)	<0.001	1.09 (1.00, 1.18)	<0.001	1.12 (1.01, 1.22)	0.009	0.92 (0.83, 1.02)	0.231

* Cox regressional anaysis was done separately for each NDHS

### Wealth index

U5MR showed a clear gradient across household wealth quintiles, since households belonging to lowest wealth quintile had highest U5MR and vice versa. Between 1996 and 2011, U5MR decreased consistently across all wealth quintiles except for fourth quintile between 2006 and 2011 (Table 3). For example, U5MR for lowest quintile was 135.7 in 1996 which had decreased to 65.3 in 2011. Though RD decreased from 59.9 in 1996 to 38.0 in 2011, it was still high between wealth sub groups. Notably between 1996 and 2011, RR, PAR had increased i.e. RR from 1.78 to 2.39 and PAR from 35.9% to 48.9%. Univariate HR was fairly consistent for wealth quintile across the four NDHSs (0.87 to 0.93) (Table 3), but Cox regression analysis showed an increasing risk of death before age of five years in all wealth groups. In 1996, HR for the lowest quintile (compared with highest wealth quintile) was 1.37 but increased to 2.54 in 2011 (Table 4). Between 1996 and 2011, both SII and RII had marginally increased for wealth sub groups (Table S2).

### Rural/urban residence

Rural areas had higher U5MR in all NDHSs, but U5MR had decreased much more in rural areas from 122.5 in 1996 to 55.8 in 2011 as compared to urban areas i.e. from 72.8 to 43.8. RD had reduced from 49.7 to 12.0 and so had RR and PAR (Table 3). Both univariate HR and HR by Cox regression did not vary much across the four NDHSs; children born in rural areas had higher risk of death before five years of age than children born in urban areas (HR ranged from 1.05 - 1.27) (Tables 3 and 4).

### Development regions

In 1996, Mid-western (143.1) and Far-western (145.7) regions had high U5MR but in 2011, Far-western region remained the highest (73.7) while in other regions U5MR was ≤50. Between 1996 and 2011, Western and Mid-western regions had achieved a substantial reduction of U5MR from 113.9 to 44.6 and 143.1 to 48.9 respectively. Though RD had decreased from 42.6 to 24.3 RR remained fairly consistent across the four surveys (1.39 to 1.65) (Table 3). HRs by univariate analysis and Cox regression analysis were inconsistent. However, in general chidren born in Far-western region (compared to eastern region) had a higher risk of death before the age of five years (HR ranged from 1.03 to 1.09) (Table 3 and 4). 

### Ecological zones

Mountainous region had the highest U5MR of 173.4 in 1996 and it reduced to 70.0 in 2011. U5MR had consistently reduced in all topographic regions and Terai (plains) region had lowest U5MR of 48.5 in 2011. U5MR decreased marginally between 2006 and 2011 in the mountains and hills (Table 3). RD had decreased from 66.5 in 1996 to 21.5 in 2011 but RD was only 1.1 in 2006. RR was similar to that of development regions and ranged from 1.02 (2006) to 1.62 (1996) (Table 3). Children born in moutainous regions (compared to plains) had a higher risk of death before the age of five years (Table 3 and 4).

## Discussion

Our secondary data analyses of complete birth histories from four NDHS showed that Nepal has achieved a steady decline in U5MR between 1991 to 2010 in terms of yearly U5MRs and three five-year intervals between four NDHSs (except between 2006 NDHS and 2011 NDHS). Projected yearly U5MR estimates indicate that Nepal is most likely to achieve MDG-4 i.e. U5MR of 54 in 2015. Despite a steady decline, measures of inequality showed that progress towards achieving MDG-4 was not evenly distributed. Wide and consistent inequalities existed according to developmental regions, ecological zones with irregular trends but inequalities for sub groups of child's sex and rural/urban residence had narrowed. Under-five children of uneducated or less educated mothers and poorest or poorer households had highest mortality in all four NDHSs. Inequalities by mother's education and wealth quintiles were widening from 1996 to 2011. 

Aggregating U5MR for longer time periods is known to smoothen the trend line, thus hiding pattern and degree of yearly change. Therefore, calculation of yearly U5MR from complete birth histories in NDHSs showed us the pattern of decline and also if a point estimate of U5MR for a given year differed from previous years or five-years average. Yearly rate of decline in our analysis (4.21%) was slightly less than that of 5.7% reported by Bhutta, Lazano and Nguyen[10,16,38]. However, U5MRs reported by Bhutta, Lazano and Nguyen were calculated up to 2008 or prior and they employed different methods to calculate U5MR. For instance, we calculated yearly estimates using direct methods, whereas Nguyen et al.[16] estimated biennial mortality rates using methods recommended by Rajaratnam et al.[39]. Such differences in yearly percentage decline may also arise from sampling errors of complex survey design despite accounting for them during analysis[40].

Our projection for the years 2011-15 showed a pattern of decline similar to the period we analysed putting Nepal on track to achieve MGD-4 which is contrary to the reported 58% probablity; a calculation based on data prior to 2005[6]. Decline of U5MR despite sampling errors encountered in NDHSs is probably true since average five-yearly decline of 19.4% exceeded 15% recommended by Karenromp et al.[40]. Projected U5MR of 54.3 in 2015 is not comparable to the projected U5MR of 31in 2015 by Nguyen et al. which was based on the data up to 2006[16]. Such a difference may have been due to non-inclusion of NDHS2011 data in which decline of U5MR was much slower than previous survey intervals. Inclusion of most recent NDHS2011 provides a more realistic estimate as it accounts for the current child mortality experience. A slight stagnation of U5MR reduction between 2006 and 2011 has been attributed to slowly declining neonatal mortality rates in developing countries[6,41,42]. U5MR decline in Nepal could be impact of interventions such as immunization, oral rehydration therapy, pneumonia treatment, and vitamin A supplementation to reduce preventable causes of child mortality such as pneumonia, diarrhoea, and measles[43]. Due to stagnation of neonatal mortality, preterm birth, neonatal sepsis and intrapartum-related conditions such as birth asphyxia may contribute to nearly 90% of neonatal deaths[42]. Specific interventions are necessary to address these preventable causes of neonatal mortality.

 A notable feature during two decades of steady decline of U5MR in Nepal is an overlapping period of decade long armed internal conflict from 1996-2006[44]. Devkota et al. have reported that 16 out of 19 health indicators had made a positive progress during the period of internal conflict and attributed the success to following reasons: Maoist rebels' non-interferance with health services, improved coordination amongst various actors, improvements in provision of health services and health programs initiated by the Government of Nepal (GoN)[13]. The GoN has been very proactive towards improving maternal and child health services by implementing several interventions: Community-based Safe Motherhood and Neonatal Health Programme and Integrated Management of childhood Illness (IMCI), free maternity services in all government health facilities, incentive program for giving birth at health facilities[18,42]. Following indicators of important child survival interventions had improved as compared to the previous surveys: skilled birth attendance, median duration of exclusive breast feeding, prevalence of undernutrition, percentage of children fully immunised, and percentage of children treated with Oral Rehydration Solutions (ORS) for diarrhoea and treated with antibiotics for pneumonia (Table S3). Improved women's education (Table 1) and declining fertility[45] as result of improved use of contraception (Table S3) might also have contributed towards reduction of U5MR in Nepal. 

Reduction of gender inequality in both absolute and relative terms is a striking feature of our analayses. Though research has shown that gender inequalities exist in Nepal and other south Asian countries[46], U5MR gender differentials were non-existent in NDHS 2011.A comparative analysis of NDHSs has shown that women's education, women's autonomy, healthcare seeking behavior and facility-based deliveries had improved over this period[45].

Absolute inequalities had decreased but relative inequalities increased in educational groups. Our results showed that 'no education' and 'primary education' sub groups had attained 50% decline in U5MR while 'secondary' and 'higher' education sub groups together achieved only about 10% decline. Increased utilisation of child survival interventions was higher among less educated mothers (Table S3) who mostly live in rural areas where child survival intervention such as community based IMCI[17,47] were implemented through FCHVs, TBAs[17,48]. Observed increase in mortality gap may also be due to saturation of preventable mortality in 'higher' and 'secondary education' sub groups. 

Despite declining U5MR in all wealth sub groups, the rich-poor gap in U5MR had widened from 1996 to 2011. Decline of U5MR was substantial in highest and middle quintiles but during post-conflict period, decline of U5MR was slower in lowest and lower wealth quintiles suggesting that income distribution may not have been equitable[49] or conflict and political instability may have caused economic downturn[21,50]. Though population living below poverty line had decreased from 41.7% in 1996 to 25.2% in 2010[51], wealth-related inequalities remained significant even after adjustment for covariates in all four NDHSs. Widening educational and wealth-related mortality gaps in Nepal are contrary to findings from Indonesia where educational mortality gap had reduced between 1982 and 1997[21]. Contextual settings of these two countries were different during differnt time periods studied. Indonesia underwent an economic boom whereas Nepal went through a period of internal conflict and political instability post-conflict period (2006 onwards). Though medical services and child survival interventions are free-of-cost, the poor are also less educated and they are known to have cultural and social barriers to access health services[52].

Urban/rural differentials had narrowed but U5MR decline in rural areas was much more than in urban areas. Rural development in terms of roads, clean energy, water and health services may have resulted in decreased morbidity and improved service utilisation of child survival interventions[53,54]. Up to 2006, Far-western and Mid-western had highest U5MR since these regions were worst affected by Maoist insurgency, had higher poverty rates, poor access to health care and emmigration[49]. A spatial modelling done after adjusting for various factors also showed that these two regions had significantly higher U5MR[55]. Between 2006 and 2011 U5MR were somewhat stagnated in all regions except Western, Mid-western regions where U5MR reduced by at least 20 (per 1000 live births). The reason for stagnation of U5MR in these regions cannot be explained from our analyses or child health service utilisation rates in these regions(Table S3) but Western and Mid-western regions being closer to the capital (Kathmandu) have easier fiscal flows and better health services or stagnated neonatal mortality[42] or unequal regional distribution of poverty since Far-western and Mid-western regions are having higher percentage of population below poverty line[51]. Hills and plains (terai) had achieved a substantial 55% decline in U5MR between 1996 and 2011 but remarkably, mountain region had achieved 59% reduction in U5MR despite having higher U5MR. Difficult terrain and inclement weather during winter months in these remote areas make provision of services difficult due to costs and retaining the health staff and make health services inaccessible due to poor transportation and communication systems[16]. Substantial decline of U5MR between 1996 and 2011 in rural areas, Farwestern, Mid-western and moutainous regions is laudable attributable to nationwide scale-up and increased utilisation of child survival interventions[12,13,17,18,47,48].

Main strength our report was analyses of four nationally respresentative NDHSs spanning two decades. NDHSs done on a reasonably large sample of women of child bearing age group are often useful resources of child mortality data in countries where vital registration systems are weak. Standardised protocols and questionnaires allow cross-country comparisons and time trend analysis. Another strength was availability of most recent survey data to forecast, if Nepal would achieve MDG-4 considering the current child mortality experience. As recommended by King et al. by calculating both absolute and relative measures we could gauge inequalities according to various sub groups[37]. In particular, HR ratios obtained by Cox  regression analyses provided a more detailed interpretation of inequalities considering the effect of specific factors and covariates. Houweling et al. have emphasised the advantage of odds ratio over the RR and RD as measures of inequalities[36]. Nevertheless, there were some limitations of using cross-sectional household survey data. The first one is about recall bias for exact date of birth, particularly in countries where mother's literacy levels are low and birth certificates for verification are unavailable for all children. In NDHS, complete birth history documents month and year only, leading to imprecise calculation of survival periods. Detection of smaller mortality reductions observed in recent NDHSs may have needed larger sample sizes owing to cluster sampling design used in NDHS. However, the samples used in all four NDHSs were not large enough leading to insufficient statitsical power for trend analysis as reported by previous studies based on DHS data for calculation of U5MR[16,21]. Therefore, projections made for the year 2015 may not be precise since about 60% of births in Nepal still take place at home without presence of a skilled attendant[17] and most of the neonatal deaths occuring within first 24 hours at home may go unreported[56].

### Policy implications

Widening inequalities by socio-economic sub groups should be addressed through non-health interventions such as women's education and poverty alleviation measures to overcome social and financial barriers for utilisation of child health services. Mountainous and Far-western regions should be given prioriy for delivering child health care services emphasising on health system strengthening particularly for essential medical supplies, recruitment and retention of health personnel. Newborn survival should be prioritised by improving community-based newborn care, shift from home births to facility births and improving the quality of antenatal care and facility births. Child survival gains achieved so far should be consolidated by strengthening existing successful child survival interventions.

## Conclusion

Projected U5MR in 2015 based on recent data was higher than the previous estimates. A consistent decline in U5MR since 1990 indicates that Nepal is most likely to achieve MDG-4. Inequalities according to gender, rural/urban residence had narrowed but educational and wealth-related inequalities may widen. Inequalities according to developmental and ecological zones though reduced in terms of absolute measures, Far-western region and mountains still lag behind in relative terms. To achieve a further reduction of U5MR, neonatal survival should be improved by scaling up recently initiated Community-Based Newborn Care Package (CB-NCP) while sustaining other successful facility-based and community-based child survival interventions with a focus on equitable access to services through 'inclusive policies' towards Far-western region and vulnerable socio-economic groups. 

## Supporting Information

Table S1 **Calculation of predicted U5MR for the period 2011 to 2015.**
(DOC)

Table S2 **Slope index of inequality and relative index of inequality in U5MR for mother's education and wealth index (data from NDHS 1996, 2001, 2006 and 2011).**
(DOC)

Table S3 **Trends in utilisation (%) of health care services according to various sub groups in Nepal during 1996(a) & 2011 (b) and absolute change (c).**
(DOC)
