# Supplementary material for: Time Trends and Inequalities of Under-Five Mortality in Nepal: A Secondary Data Analysis of Four Demographic and Health Surveys between 1996 and 2011
Source: PLoS One. 2013 Nov 4;8(11):e79818. doi: 10.1371/journal.pone.0079818 (PMC3817106; doi:10.1371/journal.pone.0079818)
Supplement: Table S1 — Calculation of predicted U5MR for the period 2011 to 2015. (DOC) [file pone.0079818.s001.doc]

**Table S1. Calculation of predicted U5MR for the period 2011 to 2015**

Iteration 0:   log likelihood = -65.479499  
Iteration 1:   log likelihood = -65.479499  
Poisson regression               Number of obs   =  19
                                                  LR chi2(1)      =      72.72
                                                  Prob > chi2     =     0.0000
 Log likelihood = -65.479499

Pseudo R2       =     0.3570
------------------------------------------------------------------------------
        u5mr |      Coef.   Std. Err.      z    P>|z|     [95% Conf. Interval]
-------------+----------------------------------------------------------------
      number |  -.0433274   .0051237    -8.46   0.000    -.0533696   -.0332852
       _cons |   4.641056   .0524901    88.42   0.000     4.538177    4.743935
------------------------------------------------------------------------------

Formula for predicted U5MR in year = e ^a+b×year^

| Year | e ^4.64 (-0.043* year)^ |
| --- | --- |
| 2011 | 64.52 |
| 2012 | 61.81 |
| 2013 | 59.20 |
| 2014 | 56.71 |
| 2015 | 54.33 |

Percentage change for each year using the formula

Annual percentage change = (e ^b^-1) × 100 (to ignore th sign)

=(0.9579-1)*100

= (-0.0421) *100

=-4.21%

For 5-yearly change by assuming that the decline was uniform

(0.8065-1)*100

=(-0.1935)*100

=-19.35 %
