# Supplementary material for: Time Trends and Inequalities of Under-Five Mortality in Nepal: A Secondary Data Analysis of Four Demographic and Health Surveys between 1996 and 2011
Source: PLoS One. 2013 Nov 4;8(11):e79818. doi: 10.1371/journal.pone.0079818 (PMC3817106; doi:10.1371/journal.pone.0079818)
Supplement: Table S3 — Trends in utilisation (%) of health care services according to various sub groups in Nepal during 1996(a) & 2011 (b) and absolute change (c). (DOC) [file pone.0079818.s003.doc]

**Table S3.** Trends in utilisation (%) of health care services according to various sub groups in Nepal during 1996(a) & 2011 (b) and absolute change (c)

|  | At least three Antenatal visits | Skilled attendance during birth | Births with two Tetanus Injections during pregnancy | Currently using any modern family planning | Measles vaccination coverage | Children with diarrhoea who did not receive any oral rehydration therapy |
| --- | --- | --- | --- | --- | --- | --- |
| **Mother’s education** | | | | | | |
| Higher | 92.1, 89, **-3.1** | 73.8, 76, **2.2** | 77.3, 86.5, **9.2** | 42.2, 34.6, **-7.6** | 92.9, 92.8, **-0.1** | 37.4, 36.7, -**0.7** |
| Secondary | 79.9, 72.4, **-7.5** | 49.8, 53.4, **3.6** | 58.6, 80.4, **21.8** | 34.3, 37.9, **3.6** | 84.9, 95.2, **10.3** |
| Primary | 55.1, 56, **0.9** | 35.5, 31.9, **-3.6** | 45.5, 66.4, **20.9** | 27.8, 40.5, **12.7** | 65, 96.3, **31.3** | 40.7, 20.2, -**20.5** |
| No education | 35.4, 42, **6.6** | 29.4, 19.4, **-9.5** | 27.1, 60.4, **33.3** | 24.5, 48.8, **24.3** | 52, 79.6, **27.6** | 53.7, 20.4, -**33.3** |
| **Wealth index*** | | | | | | |
| Highest | 84.1, 91.8, **7.7** | 57.8, 81.5, **23.7** | 78.8, 87.8, **9** | 53.9, 48.9, **-5** | 94.5, 96.1, **1.6** | 18, 18.1, -**0.1** |
| Fourth | 60.7, 77.9, **17.2** | 23, 53, **30** | 73.8, 80.5, **6.7** | 48.2, 45.3, **-2.9** | 91.3, 92.2, **0.9** | 29.1, 10.9, -**18.2** |
| Middle | 38.4, 58.3, **19.9** | 12.4, 35.9, **23.5** | 71.1, 77.7, **6.6** | 46.8, 43.3, **-3.5** | 87.4, 85.2, **-2.2** | 40.6, 25.4, -**15.2** |
| Second | 30.5, 44.7, **14.2** | 10.1, 23.7, **13.6** | 61.2, 61.7, **0.5** | 40.6, 41.1, **0.5** | 84.9, 85.2, **0.3** | 25.6, 24.2, -**1.4** |
| Lowest | 17.7, 33.3, **15.6** | 4.8, 10.7, **5.9** | 38.8, 49.8, **11** | 30.3, 35.6, **5.3** | 73.2, 86, **6.8** | 17.6, 13, -**4.6** |
| **Type of residence** | | | | | | |
| Urban | 77, 87.9, **10.9** | 58.6, 72.7, **14.1** | 48.3, 80.7, **32.4** | 45.1, 49.8, **4.7** | 77.2, 91.8, **14.6** | 38.7, 15.8, -**22.9** |
| Rural | 41.3, 54.9, **13.6** | 30.7, 32.3, **1.6** | 31.5, 68.5, **37** | 24.3, 42.1, **17.8** | 55.2, 87.6, **32.4** | 51.7, 20.9, -**30.8** |
| **Development region** | | | | | | |
| Eastern | 44, 60.7, **16.7** | 36.2, 42, **5.8** | 34, 72.2, **38.2** | 26.8, 36.2, **9.4** | 63.3, 87.9, **24.6** | 54.1, 16.4, -**37.7** |
| Central | 45.1, 56.4, **11.3** | 40.4, 35.9, **-4.5** | 40, 74.3, **34.3** | 28.6, 49.9, **21.3** | 54.8, 84.6, **29.8** | 50, 21.8, -**28.2** |
| Western | 48.9, 59.9, **11** | 23.8, 37.8, **4.6** | 32.8, 66.3, **33.5** | 25.2, 38.7, **13.5** | 56.8, 91.2, **34.4** | 46, 22.9, -**23.1** |
| Mid-western | 32.7, 53.1, **20.4** | 33.2, 28.7, **-4.5** | 26.3, 61, **34.7** | 24.2, 42.8, **18.6** | 55.8, 87.4, **31.6** | 49.8, 19.9, -**29.9** |
| Far-western | 28.6, 61.8, **33.2** | 16.6, 30.7, **14.1** | 15.7, 68.6, **52.9** | 19.6, 47.1, **27.5** | 49.1, 94.9, **45.8** | 58.7, 18.9, -**39.8** |
| **Ecological region** | | | | | | |
| Mountain | 25.7, 52.1, **26.4** | 16.8, 18.9, **2.1** | 13.8, 60.9, **47.1** | 16, 43.1, **27.1** | 49.9, 90.9, **41** | 44.9, 21.4, -**23.5** |
| Hill | 37.9, 53.2, **15.3** | 19.9, 30.4, **10.5** | 26.7, 62.4, **35.7** | 26.9, 40.6, **13.7** | 62.6, 90.4, **27.8** | 49.7, 8.1, -**41.6** |
| Terai (Plains) | 48.1, 63, **14.9** | 46.1, 42.8, **-3.3** | 40.6, 76.5, **35.9** | 26.6, 45, **18.4** | 52, 85.8, **33.8** | 53.5, 25.4, -**28.1** |

*Indicates that the change was computed between 2006 and 2011 surveys since this information was not available for either 1996 or 2001 surveys
